# Bioconversion of Untreated Corn Hull into *L*-Malic Acid by Trifunctional Xylanolytic Enzyme from *Paenibacillus curdlanolyticus* B-6 and *Acetobacter tropicalis* H-1

**DOI:** 10.4014/jmb.2105.05044

**Published:** 2021-07-15

**Authors:** Thi Bich Huong Duong, Prattana Ketbot, Paripok Phitsuwan, Rattiya Waeonukul, Chakrit Tachaapaikoon, Akihiko Kosugi, Khanok Ratanakhanokchai, Patthra Pason

**Affiliations:** 1 Division of Biochemical Technology, School of Bioresources and Technology, King Mongkut's University of Technology Thonburi, Bangkok 10150, Thailand; 2 Excellent Center of Enzyme Technology and Microbial Utilization, Pilot Plant Development and Training Institute, King Mongkut's University of Technology Thonburi, Bangkok 10150, Thailand; 3 Biological Resources and Post-harvest Division, Japan International Research Center for Agricultural Sciences, Tsukuba, Ibaraki 305-8686, Japan

**Keywords:** *Acetobactor tropicalis*, corn hull, L-malic acid, *Paenibacillus curdlanolyticus*, xylanolytic enzyme, xylose

## Abstract

*L*-Malic acid (L-MA) is widely used in food and non-food products. However, few microorganisms have been able to efficiently produce L-MA from xylose derived from lignocellulosic biomass (LB). The objective of this work is to convert LB into L-MA with the concept of a bioeconomy and environmentally friendly process. The unique trifunctional xylanolytic enzyme, PcAxy43A from *Paenibacillus curdlanolyticus* B-6, effectively hydrolyzed xylan in untreated LB, especially corn hull to xylose, in one step. Furthermore, the newly isolated, *Acetobacter tropicalis* strain H1 was able to convert high concentrations of xylose derived from corn hull into L-MA as the main product, which can be easily purified. The strain H1 successfully produced a high L-MA titer of 77.09 g/l, with a yield of 0.77 g/g and a productivity of 0.64 g/l/h from the xylose derived from corn hull. The process presented in this research is an efficient, low-cost and environmentally friendly biological process for the green production of L-MA from LB.

## Introduction

Lignocellulosic biomass (LB) is composed of three classes of polymers consisting of cellulose microfibrils embedded in a matrix of cross-linked xylan and lignin [1]. LB is abundant, readily available, affordable, sustainable, non-edible and does not pose a direct threat to food security, making it an ideal carbon sources for the production of bioproducts by microorganisms. LB is produced in enormous quantities, approximately 170–200×10^9^ tons annually worldwide [2]. It is often used for animal feed and fertilizers, however, farmers generally dispose of it by burning, which leads to air pollution and the generation of toxic fine particles, PM_2.5_, which adversely affect the respiratory systems of humans and animals [3]. An important policy strategy for reducing production costs is the use of low-cost raw materials. Consequently, converting LB into high value-added products, in addition to being extremely useful in economics, is also a smart way to deal with pollution.

Corn residue is one of the most abundant sources of LB. Corn hull, which covers the outside of the corn, is high in carbohydrates, especially hemicellulose, but low in lignin [4]. Therefore, it can be used as a potential biomass source for various biomaterial applications focused on hemicellulose utilization.

Due to the complex matrix and recalcitrant structures that are resistant to natural enzyme degradation, the bioconversion of LB is extremely challenging, and conventionally requires intensive pretreatment using physical, chemical, and/or biological methods before enzymatic hydrolysis. However, pretreatment methods have serious disadvantages, for example, high cost, use of hazardous chemicals, low chemical recovery, and pressure/corrosion resistance problems associated the equipment. Detoxification procedures due to the formation of inhibited compounds (phenolic compounds, furan derivatives and weak acids) in the post-treatment processes are necessary [5], as well as specific treatments of waste water and toxic substances before they are disposed of into the environment [6]. Furthermore, pretreatment is seen as one of the most expensive steps in converting LB to fermentable sugars, accounting for 33% of the total cost [7]. Moreover, during pretreatment, inhibitors are formed that interrupt the metabolism and growth of microorganisms, as well as enzymatic catalysis [5]. In overcoming these problems, it is good to have new technologies to facilitate the conversion of LB into fermentable sugars without pretreatment, which not only reduces costs but also eliminates unwanted compounds during the pretreatment process.

Nowadays, *L*-malic acid (L-MA) plays an increasingly important role and is gaining great interest in various industries as it can be used in a wide variety of foods as an acidifying agent and flavor enhancer for beverages, desserts, and bakery products. It also has applications in non-food products such as pharmaceuticals, metal cleaning, textile finishing and agriculture [8, 9]. L-MA has a large global market potential in the range of 200,000–600,000 metric tons/year, with a growth rate of up to 4% per year [10]. Its market value is $130 million and is expected to reach $244 million by 2024 at the current market price of US$1.75/kg [11]. Therefore, establishing an efficient method for producing L-MA is an urgent need. L-MA is produced mainly on sugars such as glucose and sucrose. The most commonly used methods for industrial production of L-MA are: (1) chemical synthesis via hydration with oil-based maleic acid/fumaric acid as the substrate to generate racemate (*DL*)-malic acid, and (2) an enzymatic route for enantiopure L-MA, involving the transformation of fumaric acid using fumarase/immobilized cells. The chemical process is associated with extreme conditions (high temperature and pressure, impurities) and expensive catalysts [8], while the enzymatic process is affected by the sensitivity of fumarase to high temperature and its inhibition by high substrate concentrations, making both methods not economically successful on a large scale [12]. Besides, their operating costs remain high and there are concerns about the environmental impact of the purification process. Currently, the bio-based economy is growing, resulting in the increasing popularity of L-MA production by microbial fermentation for two reasons: (1) the synthesis of enantiopure L-MA, which is used exclusively in pharmaceutical and polymer fields, and (2) the low production cost of L-MA can be achieved by using microorganisms and low-cost renewable materials [8]. However, until now, industrial production of microbial L-MA continues to suffer due to the low yield and the need for purification of L-MA.

The L-MA is generally produced from glucose by microorganisms, while few microorganisms can produce L-MA more efficiently from xylose. Recently, Teeravivattanakit *et al*. [13] reported that PcAxy43A, a unique weak lignin-binding trifunctional xylanolytic enzyme produced from *P. curdlanolyticus* B6, exhibits endoxylanase, β-D-xylosidase, and arabinoxylan arabinofuranohydrolase activities. This enzyme hydrolyzes xylan in raw rice straw to xylose efficiently in one step without pretreatment. Therefore, this robust enzyme was selected to produce xylose from LB, a low-cost and renewable feedstock. Moreover, we found that the newly isolated mesophilic bacterium, *Acetobacter tropicalis* strain H1 (biosafety level 1), was able to produce L-MA from xylose [14]. The objective of this research is to hydrolyze xylan present in raw LB to xylose by the trifunctional xylanolytic enzyme, PcAxy43A, for further conversion of xylose into L-MA by *A. tropicalis* strain H1. Moreover, our findings represent a contribution to the development of innovative green technology and in the effective production of L-MA from LB.

## Materials and Methods

### Preparation of Agricultural Materials

All LB, including corn cob, corn hull, Napier grass, oil palm empty fruit bunches, oil palm trunk, pineapple peel, rice husk, rice straw, sugarcane bagasse and sugarcane leaf were collected in Thailand. They were cut into small pieces using scissors, ground using an Ultra Centrifugal Mill ZM-100 (Retsch, Germany) and sifted through a 45-mesh sieve (Endecott, England). All samples were washed several times with warm distilled water to remove impurities and then oven-dried at 50°C until constant weight. Each milled LB was used as untreated material.

### Expression and Purification of Recombinant Enzyme

Purified recombinant PcAxy43A from *P. curdlanolyticus* B-6 (Accession No. KM275936), which was cloned and expressed in *Escherichia coli*, was performed by the methods as shown in our previous work [13].

### Enzyme Assay and Protein Determination

The xylanolytic enzyme activity of PcAxy43A was measured by determining the amount of xylose released from birchwood xylan (BWX) (Sigma-Aldrich, USA). The reaction mixture consisted of 0.5 ml of 1% (w/v) xylan in 50 mM sodium phosphate buffer pH 7.0 (optimum pH) and 0.1 ml of enzyme (~70 μg protein). After incubation for 10 min at 50°C, the amount of xylose was determined by the Somogyi-Nelson method [15]. One unit (U) of enzyme activity was defined as the amount of enzyme that released 1 μmol of xylose from BWX per minute under the assay conditions. The concentration of protein was measured by the Lowry method with bovine serum albumin as the standard, as described previously [13]. Each assay was conducted in triplicate.

### Determination and Analysis of Hydrolysis Products from LB by PcAxy43A

Each lignocellulosic biomass (3%, w/v) was incubated with 3 U of xylanolytic enzyme activity in 50 mM sodium phosphate buffer (pH 7.0, 50°C) at a final volume of 5 ml with shaking at 200 rpm for 5 h. The hydrolysis products were determined by the Somogyi–Nelson method, and analyzed by thin-layer chromatography (TLC) on silica gel 60 F245 plates (1.05554; 20 by 20 cm) (Merck, Germany) with a mixture of n-butanol, acetic acid, and water with portion 2:1:1 (v/v/v) as a solvent system. The sugar spots were detected by heating the plates at 100°C, with subsequent spraying with a reagent comprising 4 g of α-diphenylamine, 4 ml of aniline, 200 ml of acetone, and 30 ml of 80% (w/v) phosphoric acid [1]. Qualitative analysis of the hydrolysis products of corn hull by PcAxy43A was carried out by high-performance liquid chromatography (HPLC) (Shimadzu, Japan), using a reflective index detector (Shimadzu RID-10A) on a BP-100 Pb^2+^ carbohydrate column (Benson Polymeric, USA) [13]. Xylose (Merck) and xylo-oligosacharides (X_2_-X_6_) from Megazyme (Ireland) were used as standards. All experiments were performed in triplicate.

### Determination of Corn Hull Composition, Xylan Removal and Xylose Yield

The hemicellulose, cellulose and lignin content of the corn hull were determined using the standard laboratory analytical procedures provided by the National Renewable Energy Laboratory (NREL, USA) [16]. After the corn hull was hydrolyzed by PcAxy43A, the xylan removal and xylose yield were calculated according to the following equations:

$$\text{Xylan removal (\%)}=\frac{\text{Xylan in the untreated corn hull -Xylan in the treated corn hull}}{\text{Xylan in the untreated corn hull}}\times 100$$

$$\text{Xylose yield (\%)}=\frac{\text{Total xylose released}}{\text{Initial xylan loading}}\times 100$$

### Morphological Observation

Morphological analysis of corn hull samples was observed using a JSM-IT300LA scanning electron microscope (SEM; JEOL, Japan) operated at 10 kV. The samples were gold-coated before imaging, and then observed under variable-pressure modes at room temperature.

### Preparation of Xylose Derived from Corn Hull for L-MA Production

The xylose hydrolysis product solution derived from corn hull by the trifunctional xylanolytic enzyme PcAxy43A was freeze-dried by a bench-top freeze dryer (Labconco, USA) before being used for L-MA production.

### L-MA-Producing Strain

The L-MA-producing strain, *Acetobacter tropicalis* H1, was isolated from fermented white cabbage and identified by Duong *et al*. [14]. It was deposited in Thailand Bioresource Research Center (TBRC), Thailand, under the accession number TCBR13455. This strain was stored at –20°C in 40% (v/v) glycerol on yeast extract peptone dextrose (YPD) medium and was sub-cultured to adapt with xylose utilization on YPD medium.

### Preparation of Inoculum for L-MA Production

The *A. tropicalis* H1 strain was inoculated into a fermentation medium consisting of the following (w/v): 5%xylose; 0.3% NH_4_Cl; 0.05% KH_2_PO_4_; 0.05% MgSO_4_.7H_2_O; 0.1% yeast extraction, and 5% CaCO_3_ (functioned as the neutralizer and buffering), without pH adjustment (pH 6.95), and was used in all of the fermentation experiments. Xylose was prepared separately from other components while dry CaCO_3_ was included in each flask and then sterilized by autoclaving at 121°C, 15 psi for 20 min. Subsequently, culture was cultivated in 250-ml shake flask containing 25 ml working volume in a rotary incubator at 25°C overnight. Next, when the culture achieved an optical density at 600 nm of 0.6, then 10% (v/v) inoculum was transferred into the new fermentation medium.

### Fermentation of L-MA from Xylose Derived from Corn Hull

To investigate the effect of xylose concentration on L-MA production, the seed culture of *A. tropicalis* H1 was cultured in the 500-ml-shake flasks containing 50 ml of the fermentation medium supplements with different concentrations of xylose, which is derived from corn hull and concentrated by a freeze dryer. The shake flasks were aerobically fermented in a rotary incubator, 37°C at 200 rpm for 5 days. To determine the fermentation kinetics of L-MA, *A. tropicalis* H1 was performed at the optimum xylose concentration. All experiments were conducted in triplicated.

### Determination of Cell Biomass

The cell biomass was determined by the dry cell weight (DCW), as described previously [17]. Briefly, 1 M HCl was added into the fermentation broth with a ratio of 1:1 (v/v) to remove the excess CaCO_3_ from the culture broth. The mixture was stood for 30 min at room temperature. The pellet cells were centrifuged at 25,000 × *g* at 4°C for 10 min. Cells were washed with distilled water and collected by centrifugation and then dried till a constant weight at 105°C.

### Determination of Residual Xylose and L-MA Production

During fermentation, the concentrations of remaining xylose were measured by a high-performance liquid chromatograph (HPLC, Japan) with an Aminex-87P column (Bio-Rad, USA) and detected by a refractive index detector (Shimadzu RID-10A, Japan) at 85°C. Meanwhile, L-MA concentration was determined and quantified by HPLC using an Aminex HPX-87 H column (Bio-Rad) with a UV detector (Shimadzu, SPD-20A) at the wavelength of 210 nm, operated at 65°C. Sulfuric acid (5 mM) at a flow rate of 0.6 ml/min was used for the mobile phase of both columns.

## Results and Discussion

### Hydrolysis of Xylan in LB by PcAxy43A

Xylan is one of the major components in LB, consisting of 1,4-1inked β-D-xylopyranosyl units linked together as the main chain and the substituted residues, such as the arabinose side chain. The complete hydrolysis of xylan, particularly arabinoxylan, requires the cooperative action of multiple xylanolytic enzymes, such as endoxylanases (E.C. 3.2.1.8), β-xylosidases (E.C. 3.2.1.37), and α-*L*-arabinofuranosidases (E.C. 3.2.1.55) working in synergy [13]. The degree of xylan hydrolysis into xylose depends on the different number of individual xylanolytic enzymes and their mode of actions, and the natural form of the xylan polymer that provide a combinative interaction of multiple xylanolytic enzymes [18]. However, these enzymes are expensive, and their restrictions on specific activities and conditions cause bottlenecks [19]. Unfortunately, microorganisms that are able to simultaneously release these enzymes are rare, especially a single protein consisting of these three enzyme groups. Teeravivattanakit *et al*. [13] reported that PcAxy43A, the trifunctional xylanolytic enzyme from *P. curdlanolyticus* B-6 composed of endoxylanase, β-D-xylosidase, and arabinoxylan arabinofuranohydrolase activities hydrolyzed rice straw to xylose in one step without pretreatment. The pretreatment method is seen as one of the most expensive steps in the saccharification of LB to fermentable sugars [13]. Therefore, this enzyme can significantly reduced the production cost of xylose from LB.

As Thailand is an agricultural country, approximately 30 million tons/year of LB is produced [20]. To investigate the degradability of PcAxy43A, 10 types of LB that are readily available and large quantities were hydrolyzed by the purified PcAxy43A (Fig. S1). As shown in Table 1, the purified PcAxy43A was able to efficiently hydrolyze all of the LBs tested to produce reducing sugar without pretreatment (cut to small size only). It was found that the yield of the highest reducing sugar was obtained from corn hull (2.33 g/l), followed by corn cob (1.97 g/l), oil palm empty fruit bunches (1.95 g/l), sugarcane bagasse (1.91 g/l), sugarcane leaf (1.80 g/l), rice husk (1.66 g/l), oil palm trunk (1.59 g/l), Napier (1.39 g/l), rice straw (1.08 g/l), and pineapple peel (0.79 g/l). This result may be due to the difference in the amount of xylan and lignin present in each LB and the structure of each LB. Xylan is located in the outer layer of the plant cell-wall of LB, while lignin is the main physical obstacle in the degradation of xylan in LB by xylanolytic enzymes [7]. Therefore, among these LBs, corn hull, which yields the highest reducing sugar by PcAxy43A, was selected as a suitable feedstock for xylose production.

### Production of Xylose from Corn Hull by PcAxy43A

Substrate loading is an important factor, and influences the yield and products of enzymatic hydrolysis. Therefore, different concentrations of corn hull were optimized. The results showed that when incubated with PcAxy43A (3 U) for 5 h, the reducing sugar released from 3% (w/v) corn hull (2.33 g/l) was higher than 1% (w/v) corn hull (1.30 g/l), while it was relatively close to 5% (w/v) corn hull (2.38 g/l) and slightly lower at 7% (w/v) corn hull (2.11 g/l) (Fig. S2A). Since 1% (w/v) corn hull had too low substrate loading, it is not sufficient for enzymatic degradation. On the other hand, too high substrate concentrations can cause inhibitory processes and affect the yield and degradation rate of the enzyme [21]. In addition, since enzyme cost is an important factor in the biological economic process, enzyme dosages were also investigated. As shown in Fig. S2B, the yield of reducing sugar increased with increasing enzyme dosage. However, at the optimum hydrolysis time (5 h), the highest reducing sugar reached 2.48 g/l at 4 U of PcAxy43A, which is quite close to the 3 U (2.33 g/l). Therefore, 3% (w/v) corn hull and 3 U of PcAxy43A at 5 h were selected for further experiments.

**Fig. 1:**
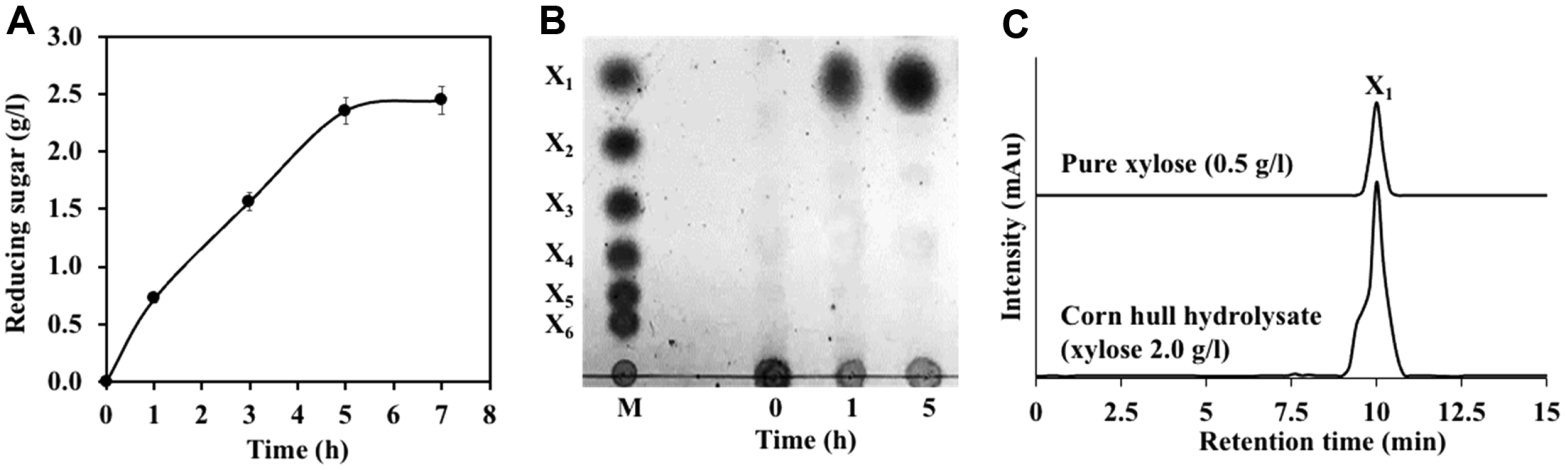
Analysis of hydrolysis products of corn hull (3%, w/v) by 3 U PcAxy43A. (**A**) Time course of reducing sugar produced from corn hull by PcAxy43A, (**B**) TLC separation of products released from corn hull by PcAxy43A. Lane M: standard xylose to xylohexaose: X_1_, xylose; X_2_, xylobiose; X_3_, xylotriose; X_4_, xylotetraose; X_5_, xylopentaose; X_6_, xylohexaose. Corn hull (3%, w/v) was incubated with 3 U PcAxy43A in 50 mM phosphate buffer, pH 7.0 at 50°C with shaking at 200 rpm, (**C**) HPLC profiles of corn hull (3%, w/v) hydrolysis product using 3 U PcAxy43A compared to the pure xylose. Experiments were performed in triplicate, while the error bars represent ± SDs (*n* = 3).

The reducing sugar profile of the hydrolysis products showed that when using 3% (w/v) corn hull and 3 U of PcAxy43A, the amount of reducing sugar was increased rapidly over a 5-h period with a maximum of 2.33 g/l, yielding 27.1% of xylan conversion. After that, the reducing sugar content remains constant (Fig. 1A). It is possible that due to the limited amount of xylan left in the corn hull, the xylanolytic enzyme was inhibited by its hydrolysis products [22]. Moreover, Teeravivattanakit *et al*. [13] reported that xylan in LB has two forms, the free and linked xylans. Free xylan can be easily hydrolyzed by the xylanolytic enzyme, while the linked xylan which is attached to lignin, through ether and ester linkages, and xylan linked with cellulose microfibrils through hydrogen bonds could not. Therefore, the xylanolytic enzyme PcAxy43A could hydrolyze only the free xylan present in corn hull but not for the linked xylan. However, increasing the xylan conversion yield, while protecting the environment, can be achieved through mild physical methods such as warming or autoclaving [7].

As shown in Fig. 1B, between 1 and 5 h only xylose was detected as a single product. However, analysis of corn hull (3% w/v) hydrolysis product with 3 U PcAxy43A at 5 h by HPLC revealed that xylose was the main product (~88% purity) at a concentration of 2.0 g/l (Fig. 1C). This result indicates that endoxylanase and β-xylosidase of PcAxy43A work well together to hydrolyze xylan in corn hull to xylose. PcAxy43A produced mainly xylose, but it did not produce arabinose, in which arabinose is one component of arabinoxylan of corn hull. This may be because the arabinose in arabinoxylan of corn hull is linked to lignin and/or cellulose [7]. Teeravivattanakit *et al*.[13] also found that the trifunctional xylanolytic enzyme, PxAxy43A, has the ability to hydrolyze xylan in rice straw to xylose in one step. Ye *et al*. [23] reported that the alkaline-pretreated corn stover was hydrolyzed to xylose by a xylanolytic enzyme from *Penicillium oxalicum*; however, it released only 0.05 g/l of xylose in 24 h. The resulting xylose concentration was much lower and took much longer to hydrolyze compared to our study. A wide variety of value-added products can be produced directly from xylose, such as organic acids, xylitol, bioethanol, biobutanol, 1,3-propanediol, 2,3-butanediol, biopolymers, amino acids, and single-cell protein [24].

### Morphology of Corn Hull after Hydrolysis with PcAxy43A

Xylan is usually located on the outer layer of LB [1]. Corn hull is an interesting biomass for xylose production due to its high hemicellulose content and low lignin content [4]. After the corn hull was hydrolyzed by PcAxy43A (5 h), the hemicellulose content (mainly xylan) [25] was significantly reduced from 28.66% to 21.96%, with a 23.38% xylan removal, while the cellulose and lignin contents increased by 10.32% and 14.60%, respectively (Table 2).

**Fig. 2:**
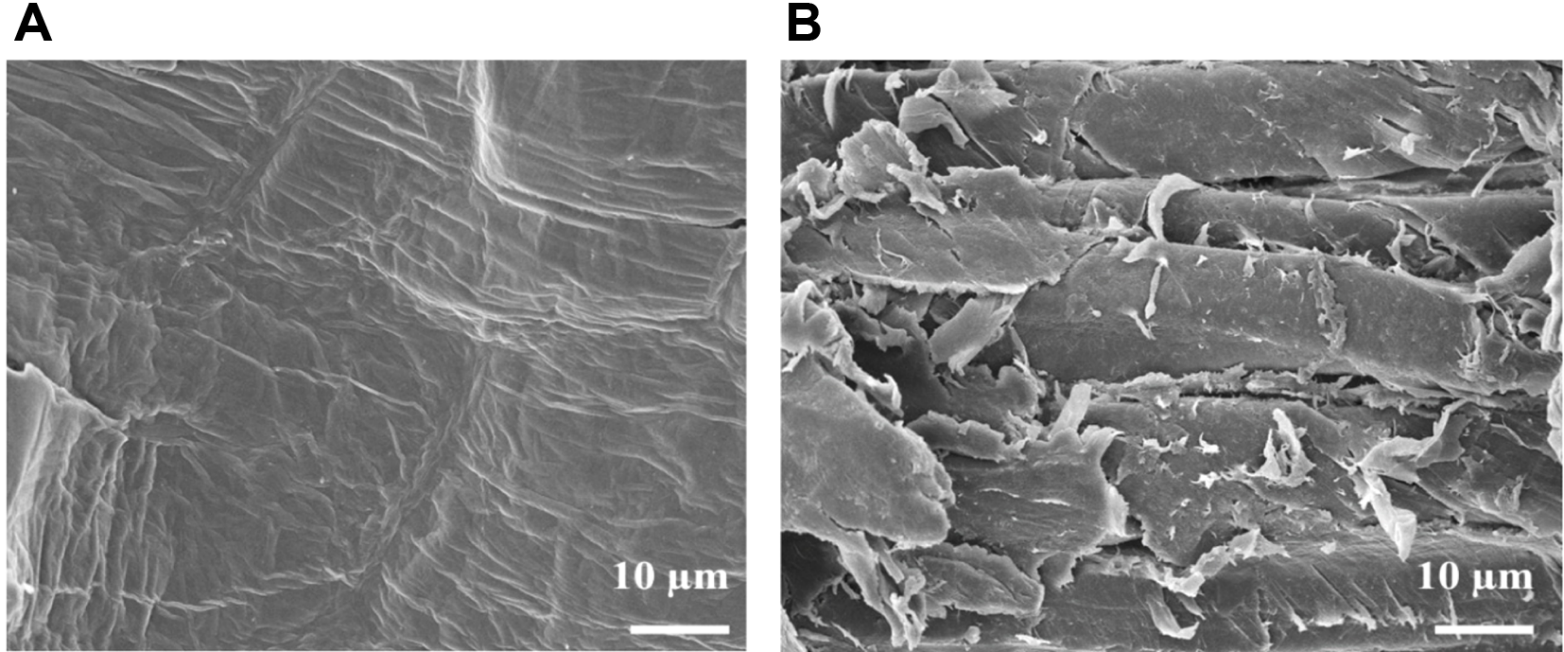
SEM of corn hull surface structures at ×1,000 magnification. (**A**) Untreated corn hull, (**B**) treated corn hull after incubation with 3 U PcAxy43A for 5 h.

To verify that xylan in the corn hull was actually removed, SEM was used to observe the surface structure of the corn hull samples. The untreated corn hull showed a rigid, smooth and well-ordered structure (Fig. 2A). As expected, after partial xylan removal, xylan in the fibrous surface of the treated corn hull was destroyed by the action of PcAxy43A, causing morphological and surface structure changes (Fig. 2B). This result suggests that PcAxy43A could attack and hydrolyze xylan in the corn hull at the molecular level. Baramee *et al*. [26] reported that after rice straw was disrupted by a cellulase-free xylanolytic enzyme from *Bacillus firmus* K-1, the morphological and surface structure of the rice straw changes. The extensive damage to the fiber by the xylanolytic enzyme causes the structure to loosen, swell, and create large porosities on the surface of rice straw. Moreover, after xylan removal, the corn hull residue can be used as biologically treated corn hull to enhance the saccharification of cellulose to glucose by cellulases, while the higher lignin content of corn hull residue makes it easier to be used as a raw material for modern biotechnology products, with antitumor, antiviral, antioxidant and antimicrobial activities [27] compared to untreated corn hull. Therefore, PcAxy43A not only produced xylose from the xylan contained in LB, but is also considered as a green enzymatic pretreatment to remove xylan from LB for further use.

### Conversion of Xylose Derived from Corn Hull to L-MA by *A. tropicalis* H1

The L-MA-producing microorganisms can be divided into two groups: fungal species of the *Aspergillus*, *Rhizopus*, *Ustilago*, *Zygosaccharomyces*, *Schizophyllum*, *Penicillium*, and *Aureobasidium* genera [8, 9], and bacterial such as *Bacillus subtilis* and *Bacillus licheniformis* [28] and *Acetobacter pasteurianus* A11-2 [29]. Some microorganisms produce high amounts of L-MA, for example, *Aspergillus flavus* produces 113 g/l of L-MA. However, since aflatoxin is detected, this strain cannot be used in industry [30]. Therefore, the production of L-MA requires highly safe microorganisms, especially for food products.

Although many microorganisms have been reported to produce L-MA from glucose, only a few microorganisms can efficiently use xylose as a carbon source to produce L-MA because there are no specific transporters and lack of a cocktail enzyme involved in xylose conversion to L-MA [31], which leads to the loss of large amounts of pentose sugars when using LBs as raw materials. Therefore, to solve this problem, many researchers are trying to create genetically modified strains such as recombinants *E. coli*, *Aspergillus oryzae*, and *Saccharomyces cerevisiae*. However, in this approach, L-MA yield was low due to the obscure L-MA synthesis regulation genes or unstable plasmids throughout fermentation [32]. Until now, few microorganisms have been reported to produce L-MA from xylose, the xylan subunit present in LB. The newly isolated *Acetobacter tropicalis* H1, rated as biosafety level 1 class, can produce L-MA from a number of sugars, especially with xylose as a carbon source [14]. Therefore, *A. tropicalis* H1 was selected to convert xylose derived from LB, which are renewable and low-cost feedstock into L-MA.

**Fig. 3:**
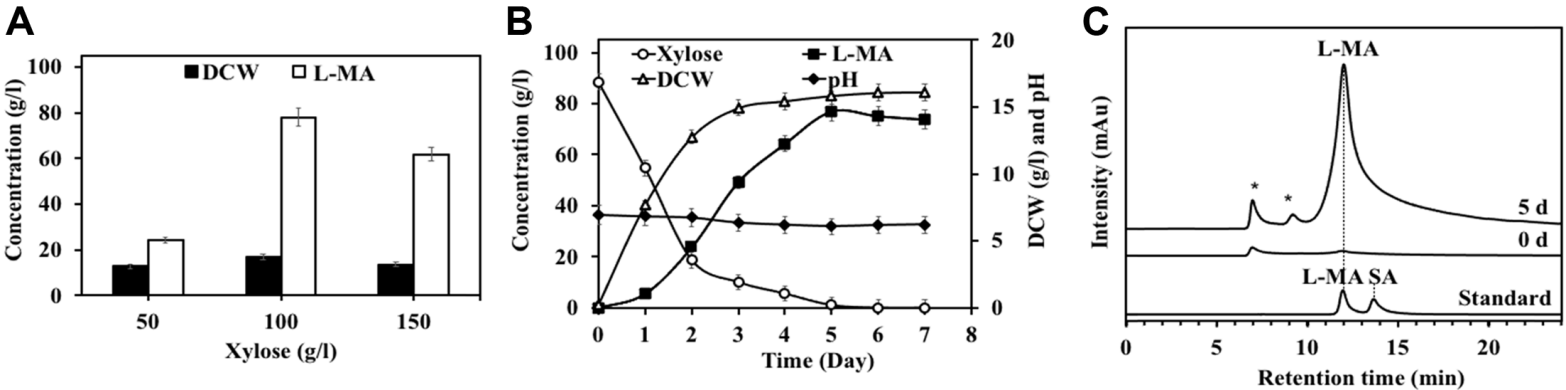
The L-MA produced from xylose derived from corn hull by *A. tropicalis* H1. (**A**) Effect of initial xylose concentration (from corn hull) on growth and L-MA production on day 5, (**B**) Time courses of batch fermentation of DCW, LMA production, xylose consumption and pH during fermentation at 100 g/l xylose. Each experiment was performed in triplicate. The error bars represent ± SDs (*n* = 3), (**C**) HPLC analysis of L-MA acid produced from xylose. L-MA, L-malic acid, and SA, succinic acid was used as standards. * refers to other organic acids.

L-MA is known as an intermediate in the tricarboxylic acid cycle (TCA cycle) and produces a high titer when using high sugar concentrations [33]. Therefore, the effect of xylose derived from corn hull at high concentration on L-MA production was conducted. As shown in Fig. 3A, on day 5 of fermentation, at 100 g/l xylose from corn hull, the maximum DCW (16.91 g/l) and L-MA at a titer of 78.12 g/l were produced in the culture broth, which was about 3 times of L-MA higher than the productivity at the initial xylose concentration of 50 g/l (25.92 g/l). On the other hand, too high concentrations of the xylose (150 g/l) resulted in lower DCW (17.88 g/l) and L-MA production (62.87 g/l) compared to 100 g/l xylose from corn hull. The inhibition phenomena can be caused by excessive osmotic stress and/or too high substrate concentrations, which hinder the growth of L-MA-producing microorganisms [34]. Therefore, the initial xylose concentration of 100 g/l (~88% purity) was selected for further experiments. These results suggest that *A. tropicalis* H1 is highly capable of utilizing high concentrations of xylose to produce L-MA. It is possible that this strain may carry specific transporter(s) and enzyme system involved in the conversion of xylose into L-MA, and may be due to enzyme deficiency involved in the subsequent conversion of L-MA to other products, which cannot be explained at the moment. However, Komesu *et al*. [35] reported that xylose-fermenting bacteria initially take up xylose into the cells through pentose phosphate pathway (PPP) via a specific sugar transporter, after that xylose was converted to *D*-xylulose 5-phosphate as a precursor intermediate of the PPP by xylose isomerase and xylulose kinase. To understand the pathways involved in the synthesis of L-MA from xylose in the strain H1, we plan to conduct future studies by investigating the enzymes involved in the conversion of xylose to L-MA, transcription of the genes encoding these enzymes via adaptation of the gene expression level [36] and metabolic flux identification of this strain [37]. In addition, to optimize the production of L-MA to make the process more economical, we plan to optimize the cultivation conditions such as nitrogen source, CaCO_3_ concentration, and aeration rate.

To understand the fermentation kinetics of L-MA produced from xylose derived from non-pretreatment milled corn hull by *A. tropicalis* H1, the relationship between the cell growth (DCW), L-MA production, xylose consumption, and pH were carried out in a rotary incubator with the optimal initial xylose concentration. Fig. 3B shows that since we used fresh and active inoculum, the cells could grow without the lag phase. Between day 0 and day 3 (logarithmic phase), xylose was consumed rapidly while DCW and L-MA were increased to 14.98 g/l and 49.29 g/l, respectively. After day 3 (stationary phase), and possibly due to xylose limitation, DCW was quite constant (~15 g/l). The L-MA was produced rapidly from xylose derived from corn hull (the purity of the xylose is about 88%, Fig. 1C) by *A. tropicalis* H1 and reached its maximum peak on day 5, measuring 77.09 g/l of L-MA, corresponding to a yield of 0.77 g/g L-MA and productivity of 0.64 g/l/h (Fig. 3B). Meanwhile, the yield of L-MA production from pure xylose by strain H1, corresponding to L-MA 87.53 g/l, yielding of 0.88 g/g L-MA, and productivity of 0.73 g/l/h (Table 3). These results indicated that the titer, yield, and productivity of L-MA from xylose derived from corn hull by strain H1 were slightly lower than pure xylose due to lower xylose purity. Moreover, these results suggest that *A. tropicalis* H1 produced L-MA during the logarithmic phase and reached maximum at the stationary phase. It is noteworthy that L-MA production decreased slightly from day 5 to day 7, which may be due to the limitations of xylose in utilization under the late stationary growth phase, and the possibility to convert small amounts of L-MA to other metabolic intermediates under the TCA cycle. Prabhu *et al*.[38] reported that after the maximum production of succinic acid from xylose by *Yarrowia lipolytica*, the succinic acid is reduced because it is converted to other by-products. On the other hand, the pH decreased slightly from 6.95 to 6.07 due to the formation of L-MA. The pH of the culture medium did not change significantly due to CaCO_3_ in addition to being responsible for the supply of CO_2_ for carboxylation of pyruvate, which is a key factor in L-MA production and also acts as a buffer [39].

**Fig. 4:**
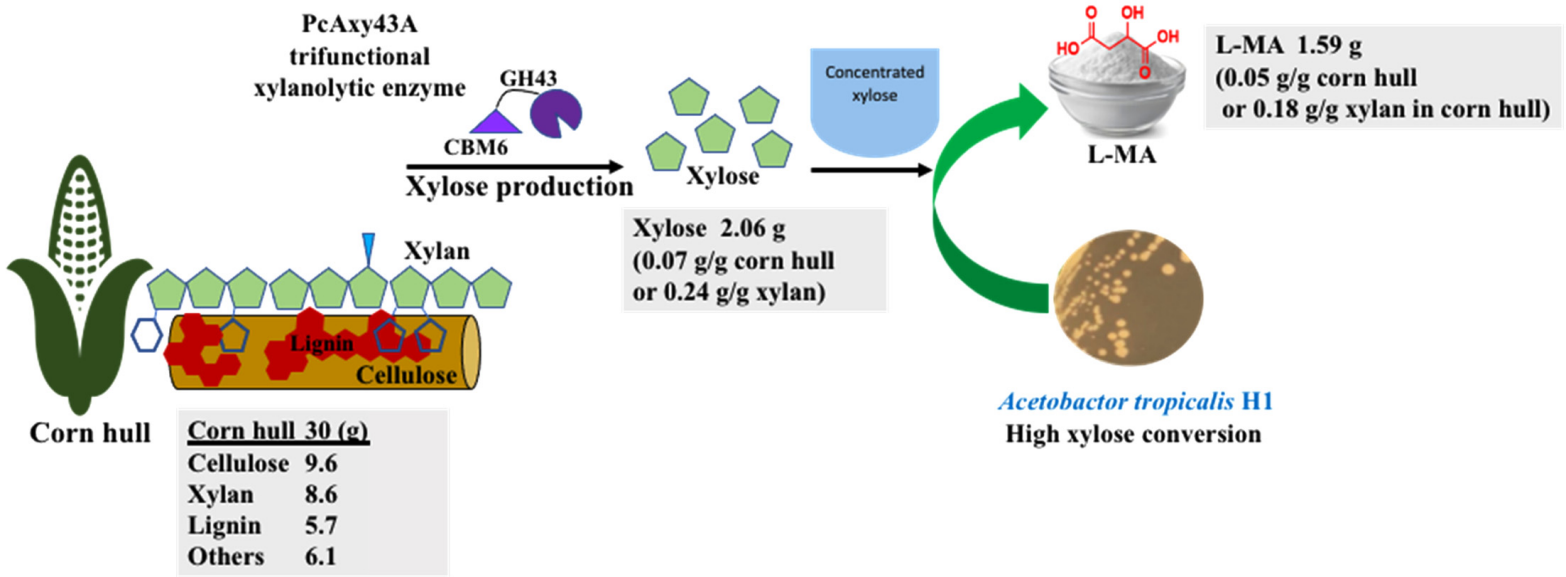
Flow chart of the overall bioconversion process of untreated corn hull into L-MA by trifunctional xylanolytic enzyme from *P. curdlanolyticus* B-6 and *A. tropicalis* H-1.

According to conventional fermentation, L-MA-producing microorganisms produced approximately 12-80% of L-MA, while others by-products such as fumaric acid, citric acid and others are also produced. For example, *Schizophyllum commune* 9384 strain produced malic acid (~12.4%), oxalic acid (7.6%), fumaric (0.3%) and succinic (0.5%) from pure xylose [40], *T. fusca* muC produced malic acid (~50%) with succinic acid (~25%) and others (~25%) from milled corn stover [10], while *Rhizopus delemar* HF-119 accumulated approximately 60 g/l of malic acid (~76%) with fumaric acid (~10%), succinic acid (~3%), and ethanol (~11%) from corn straw hydrolysate [37]. These by-products are difficult to separate from L-MA even after following metabolic engineering strategy [41]. Dai *et al*. [32] reported that the production of L-MA remains challenging and difficult to reach commercially, because in the purification of L-MA, more than 50% of the total cost is used to separate other organic acids, such as succinic, fumaric, and acetic acids. The solution is to find new indigenous strains or metabolic-engineered strains capable of producing L-MA as a main product and develop a new low-cost and efficient L-MA purification process. Fig. 3C shows that at the optimum conditions, *A. tropicalis* H1 produced L-MA as the main product (approximately 80%), along with small amounts of other organic acids including succinic (8%), citric (4%), oxalic (3%), formic (3%) and acetic acid (2%) (Table S1). In addition, we found that L-MA production could be enhanced by using a fresh and active inoculum for the cells growing without the lag phase that directly utilized xylose to produce high metabolite product titer (Fig. 3B). Furthermore, the process optimization of *A. tropicalis* using a mineral salt medium containing xylose at pH 7.0 and 37ºC with CaCO_3_ as a neutralizing agent and CO_2_ supply resulted in high growth rates and accumulation of malic acid with a few by-products (Fig. 3). Moreover, *A. tropicalis* H-1 did not produce color in the culture broth while it remained white on LB agar plate, indicating no dark pigment accumulation, which is different from some L-MA-producing strains, such as *Aureobasidium pullulans* GXZ-6, which secreted impurity (melanin) during L-MA production, making it difficult to recover and purify [42]. Therefore, L-MA produced from xylose derived from corn hull by *A. tropicalis* H-1 can be easily purified and will be particularly helpful in reducing costs in the purification process and providing a novel approach for L-MA production. This biological process is efficient, low cost and environmentally friendly for the green production of L-MA from corn hull. The overall bioconversion of untreated corn hull into L-MA by trifunctional xylanolytic enzyme from *P. curdlanolyticus* B-6 and *A. tropicalis* H-1 in this study is shown in Fig. 4. From 30 g of corn hull, 2.06 g of xylose was obtained with a yield of 6.87% then converted to 1.59 g of L-MA with a yield of 5.3%.

Because of the complex matrix and recalcitrant structures, most xylose based-LB requires a pretreatment step such as steam explosion, acid hydrolysis, or the organosolv process [8]. Many successes have been reported in the production of L-MA from various treated LBs such as barley straw [34], beech wood hemicellulose fraction [30], corn cob hydrolysate [43], corn fiber hydrolysate [44], corn straw hydrolysate [37], and wheat straw [44] by microorganisms under various processes with malic titers ranging from 5.8–120.5 g/l, yields of 0.30–0.96 g/g and productivity of 0.03–2.01 g/l/h (Table 3). The *A. tropicalis* H1 strain produced high L-MA concentration (77.09 g/l) using xylose derived from raw corn hull as a carbon source, yielding 0.77 g/g, and a productivity of 0.64 g/l/h. Thus, the strain H1 produced L-MA from untreated corn hull in the range of L-MA production from various LBs by microorganisms. While few studies have focused on the production of L-MA from untreated LB, for example, Deng *et al*. [10] reported that the engineered *Thermobifida fusca* muC-16 strain could directly convert non-pretreatment milled corn stover into L-MA with a titer of 21.47 g/l. However, the muC-16 strain produced ~3.6 times lower L-MA than *A. tropicalis* H1 (77.09 g/l).

Compared to the aforementioned strains, the safety strain *A. tropicalis* H1 had better positive properties for L-MA production. First, the strain H1 is capable of high yielding and high productivity of L-MA from untreated corn hull. Second, strain H1 did not generate pigment during fermentation, making it easier to recover and purify. Unlike strain H1, some L-MA-producing strains, such as *A. pullulans* GXZ-6, secreted melanin during L-MA production [42]. Third, at the optimum conditions, strain H1 produced few by-products during the production of L-MA (Fig. 3C), which simplifies the recovery and purification processes and leads to lower costs. Therefore, L-MA production from xylose derived from raw corn hull by *A. tropicalis* H1 has high potential for L-MA production.

In conclusion, the most commonly researched carbon source for microbial L-MA production is glucose, while only certain microorganisms can effectively convert xylose to L-MA. In this study, xylose was produced from corn hull without pretreatment in one step using a unique trifunctional xylanolytic enzyme from *P. curdlanolyticus* B-6. Thereafter, *A. tropicalis* H1, at biosafety level 1 was able to convert the high xylose concentration to high purity L-MA. This work demonstrates a good strategy to present a good method for producing L-MA from LB, which is an attractive, economical, and environmentally friendly biological process.

## Supplemental Materials

Supplementary data for this paper are available on-line only at http://jmb.or.kr.

## Figures and Tables

**Table 1:** Reducing sugar released from various raw LB by PcAxy43A.

Substrate	Reducing sugar (g/l)
Corn cob	1.97 ± 0.11
Corn hull	2.33 ± 0.13
Napier	1.39 ± 0.20
Oil palm empty fruit bunches	1.95 ± 0.15
Oil palm trunk	1.59 ± 0.30
Pineapple peel	0.79 ± 0.13
Rice husk	1.66 ± 0.10
Rice straw	1.08 ± 0.11
Sugarcane bagasse	1.91 ± 0.12
Sugarcane leaf	1.80 ± 0.23

The reactions consisted of 3% (w/v) of each LB and xylanolytic enzyme (3 U) in 50 M of phosphate buffer pH 7.0 and incubation at 50°C, shaken at 200 rpm for 5 h. Experiments were performed in triplicate. The error bars represent ± SDs (*n* = 3).

**Table 2:** Composition of corn hull before and after hydrolyzed by PcAxy43A and xylan removal (%).

Sample		Composition (% dry weight)		Xylan removal (%)

	Xylan	Cellulose	Lignin	
Untreated corn hull	28.66 ± 0.64	32.06 ± 0.79	19.11 ± 0.84	-
Treated corn hull	21.96 ± 0.65	35.37 ± 1.05	21.90 ± 0.65	23.38 ± 1.21

This experiment was performed in triplicate. The error bars represent ± SDs (*n* = 3).

**Table 3:** Comparison of L-MA production from various LB by microorganisms.

Substrate	Pretreatment	Substrate concentration	Microorganism	Process mode	L-MA (g/l)	Yield (g/g)	Productivity (g/l/h)	Reference
Corn hull hydrolysate	Non- pretreatment (Milled into small particles)	100 g/l (88 g/l xylose)	*A. tropicalis* H1	15-ml shake flask, batch, 120 h, 37ºC	77.09	0.77	0.64	This study
Xylose	-	100 g/l	*A. tropicalis* H1	15-ml shake flask, batch, 120 h, 37ºC	87.53	0.88	0.73	This study
Milled corn stover	Non-pretreatment (Milled into small particles)	50 g/l	*Thermobifida fusca* muC-16	3-l bioreactor, batch (120 h), 55ºC	21.47	0.43	0.18	[10]
Barley straw	Acid	95 g/l (56 g/l glucose, 33 g/l xylose and 6 g/l arabinose)	*Aureobasidium pullulans* Y-2311-1	125-ml shake flask, batch (288 h), 25ºC	43.54	0.30	0.48	[34]
Beech wood hemicellulose hydrolysate	Organosolv	99.5 g/l	*Aspergillus oryzae* DSM 1863	2-l bioreactor, batch (168 h), 32ºC	5.8	-	0.03	[30]
Corncob hydrolysate	Acid	150 g/l (96 g/l glucose and 54 g/l xylose) *A. pullulans* CCTCC M2012223	5-l bioreactor, batch (864 h), 25ºC	38.6	0.30	0.40	[43]
Corn fiber hydrolysate	Chemical (alkaline + H_2_O_2_)	50 g/l	*A. pullulans* NRRL 50383	50-ml shake flask, batch (168 h), 25ºC	11.6	-	0.07	[44]
Corn straw hydrolysate	Stream-steam-explosion jar and dilute acid	125 g/l (100 g glucose and 25 g xylose)	*Rhizopus delemar* HF-121	3-l bioreactor, batch (60 h), 30ºC	120.5	0.96	2.01	[37]
Wheat straw hydrolysate	Chemical (alkaline + H_2_O_2_)	50 g/l	*A. pullulans* NRRL 50383	50-ml bioreactor, fed- batch (168 h), 25ºC	27	-	0.16	[44]
