# Supplementary material for: Bioconversion of Untreated Corn Hull into L-Malic Acid by Trifunctional Xylanolytic Enzyme from Paenibacillus curdlanolyticus B-6 and Acetobacter tropicalis H-1
Source: J Microbiol Biotechnol. 2021 Jul 15;31(9):1262–71. doi: 10.4014/jmb.2105.05044 (PMC9705945; doi:10.4014/jmb.2105.05044)
Supplement: Supplementary file 1 [file jmb-31-9-1262-supple.pdf]

J. Microbiol. Biotechnol.
https://doi.org/10.4014/jmb.2105.05044

Supplemental Table and Figure

Table S1. Organic acid composition produced by *Acetobacter tropicalis* H1 during fermentation using xylose derived from corn hull as a carbon source for 5 days.

**Organic acid**	**Peak area**	**Composition (%)**
Acetic	204720	2
Citric	464771	4
Formic	304106	3
Malic	8803529	80
Oxalic	330333	3
Succinic	870456	8

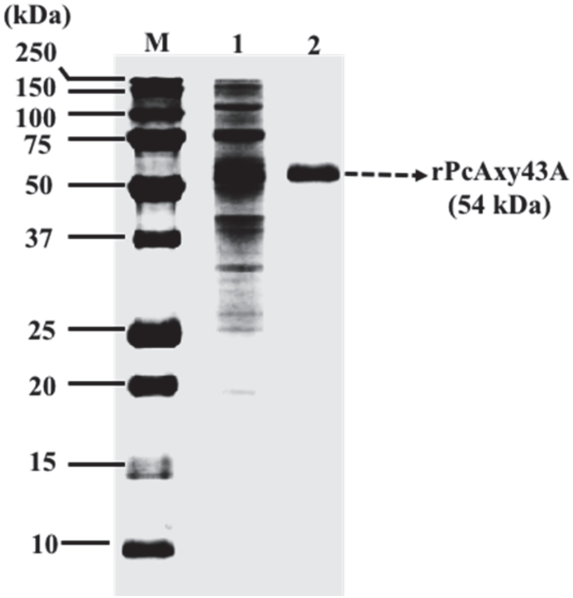

Fig. S1. SDS-PAGE analysis of purified recombinant protein. Lane M, protein marker; Lane 1, cell-free extract of PcAxy43A; Lane 2 purified PcAxy43A.

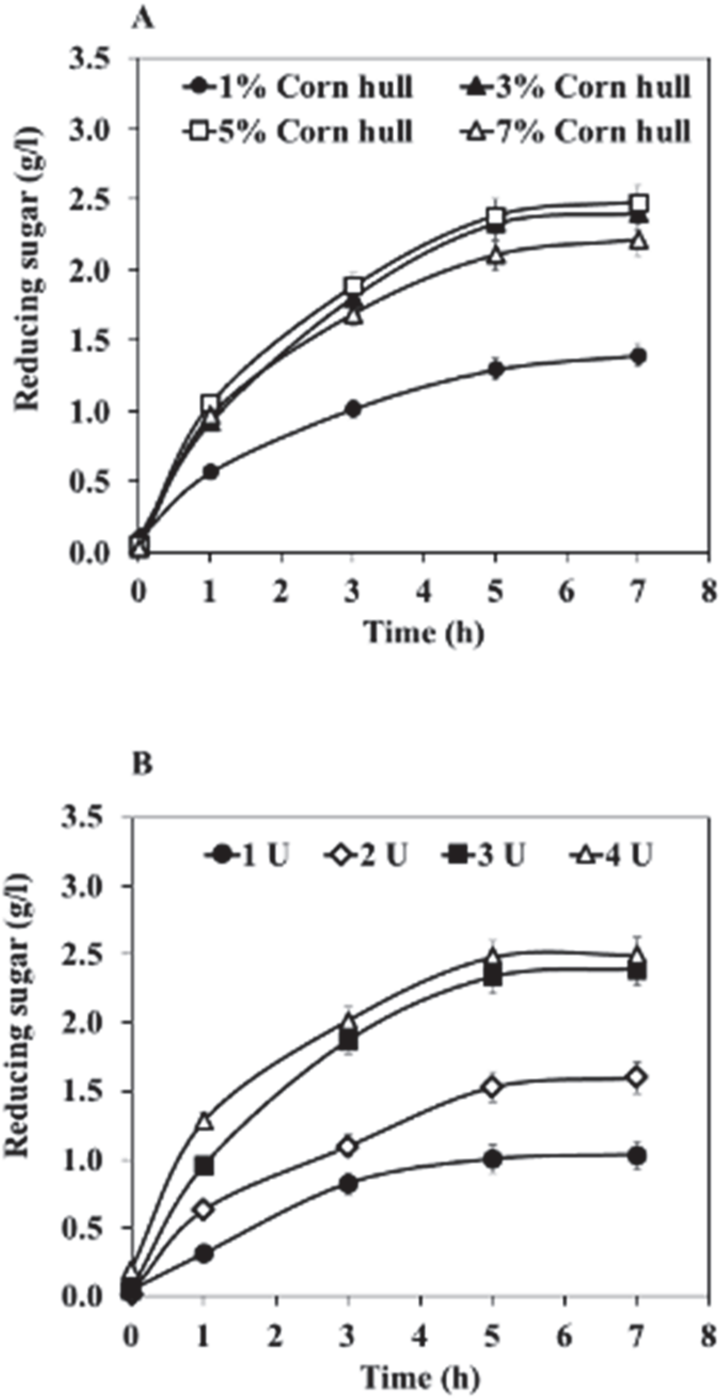

Fig. S2. Saccharification of corn hull by PcAxy43A. **(A)** Optimizing the loading of various substrate concentrations with the PcAxy43A (3 U). **(B)** Optimizing the loading of various enzyme units with (3%, w/v) corn hull. The reactions were carried out in 50 M sodium phosphate buffer and incubated at 50°C with shaking at 200 rpm. Each experiment was performed in triplicate. The error bars represent ± standard deviations (n = 3).
